# Comparative Analysis of Chemical Activators and Expansive Agents for Aeolian Sand Stabilization Using Industrial Solid Waste-Based Geopolymers

**DOI:** 10.3390/gels11090713

**Published:** 2025-09-04

**Authors:** Zilu Xie, Zengzhen Qian, Xianlong Lu, Hao Wang, Phatyoufy Lai

**Affiliations:** 1 School of Engineering and Technology, China University of Geosciences, No. 29 Xueyuan Road, Haidian District, Beijing 100083, China; 3002220008@email.cugb.edu.cn (Z.X.); 2102230104@email.cugb.edu.cn (H.W.); 9102240003@email.cugb.edu.cn (P.L.); 2 State Grid Electric Power Engineering Research Institute Co., Ltd., No. 8 Kaiyang Road, Fengtai District, Beijing 100069, China; luxianlong@163.com

**Keywords:** aeolian sand, chemical activators, expansive agents, industrial solid wastes-based geopolymer

## Abstract

Aeolian sand is the primary geological material for construction in desert regions, and its stabilization with industrial solid wastes-based geopolymer (ISWG) provides an eco-friendly treatment replacing cement. This study comparatively investigated the enhancement effects of chemical activators and expansive agents on compressive strength of aeolian sand stabilized by ISWG (ASIG). Three chemical activators—NaOH, Ca(OH)_2_, and CaCl_2_—along with two expansive agents—desulfurized gypsum and bentonite—were considered. Through X-ray diffraction, thermogravimetric analysis, scanning electron microscopy, mercury intrusion porosimetry and pH values tests, the enhancement mechanisms of the additives on ASIG were elucidated. Results demonstrate that the expansive agent exhibits significantly superior strengthening effects on ASIG compared to the widely applied chemical activators. Chemical activators promoted ISWs dissolution and hydration product synthesis, thereby densifying the hydration product matrix but concurrently enlarged interparticle pores. Desulfurized gypsum incorporation induced morphological changes in ettringite, and excessive desulfurized gypsum generated substantial ettringite that disrupted gel matrix. In contrast, bentonite demonstrated superior pore-filling efficacy while densifying gel matrix through a compaction effect. These findings highlight bentonite superior compatibility with the unique microstructure of aeolian sand compared to conventional alkaline activators or expansive agents, and better effectiveness in enhancing the strength of ASIG.

## 1. Introduction

Arid and semi-arid desert regions account for 12% of the global terrestrial area, and are projected to expand to 12.9–13.7% by the end of the 21st century [1]. These deserts are predominantly distributed across vast areas of North Africa, the Middle East, North America, and Central Asia. As human urban areas and activities continue to expand, exemplified by strategic initiatives such as the Belt and Road Initiative [2] and Vision 2030 [3] that encompass extensive desert territories, inevitably necessitate the construction of future infrastructure development, including buildings, highways, and industrial facilities in desert environments where aeolian sand serves as the primary foundation material. However, as a special soil type shaped by unique geological processes and climatic conditions, aeolian sand exhibits inherent characteristics of low natural moisture content, loose structure, and absence of interparticle cementation under natural conditions, resulting in poor mechanical properties [4,5,6]. Although numerous studies have utilized aeolian sand as aggregate in aeolian sand-based concrete and subgrade fillers [7,8,9,10,11], achieving valuable outcomes that partially address material shortages for desert construction, the utilization of aeolian sand foundations—particularly critical for high-rise buildings and large-span structures in desert regions—poses significant challenges to structural stability and reliability, thereby constraining urban development.

In situ stabilization using gels materials has been widely recognized as a more cost-effective and efficient alternative to soil replacement for improving aeolian sand foundations. Ordinary Portland Cement (OPC), as the most commonly used inorganic stabilizer, has been extensively applied and studied for aeolian sand stabilization [12,13,14]. However, production of OPC is associated with substantial carbon emissions from limestone calcination and fossil fuel combustion, rendering it unsustainable within the global context of carbon peaking and neutrality goals [15]. Research in past decade has proposed utilizing industrial solid wastes (ISWs) such as ground granulated blast furnace slag (GGBFS), steel slag (SS), red mud (RM), fly ash (FA), and carbide slag (CS) to develop geopolymer based on ISWs (ISWG) for stabilizing soils with poor engineering properties [16,17,18,19,20]. These ISWs contain substantial quantities of reactive dicalcium silicate (C_2_S), tricalcium silicate (C_3_S), and calcium aluminate (C_2_A), which undergo hydration reactions to form gels that connect soil particles. However, ISWs may exhibit low hydration reactivity due to the high crystallinity and strong chemical bonds in oxide caused by slow cooling rate during manufacturing and production environment [21,22], which adversely affects the quantity of hydration products, thereby restricting the strength development of the stabilized soil.

The strength of soil stabilized by geopolymer is widely acknowledged to primarily originate from hydrated calcium aluminosilicate (C-(A)-S-H) gel synthesized in the presence of activators such as Ca(OH)_2_ [16,17,18,19,20,23,24,25,26], which bonds soil particles and fills interparticle pores densely. The mechanism remains applicable to aeolian sand stabilized by ISWG (ASIG). The gel component can be augmented through chemical and mechanical activation of ISWs, which enhances their hydration reactivity and consequently elevates the yield of hydration products [24,27,28]. Chemical activation, mechanical grinding, and high-temperature calcination are currently prevalent activation methods for ISWs. Although their activation mechanisms differ, they share the common objective of enhancing the strength of ISWG. However, chemical activation is regarded as a more effective, cost-efficient, and eco-friendly approach since it does not require energy-intensive equipment such as ball mills or calcination furnaces, and it has been extensively investigated [22,29,30,31]. The activation mechanism of alkaline activators is predominantly ascribed as the ability of their ionized OH^−^ to cleave covalent bonds within ISWs, including Si-O-Si, Si-O-Al, and Al-O-Al bonds. The liberated ions subsequently participate in polymerization reactions, leading to the formation of additional hydration products [22,31,32,33]. On the other hand, studies have demonstrated that chloride salts such as CaCl_2_ can react with aluminum-bearing phases in gels systems to synthesize hydrocalumite also known as Friedel salt (3CaO·Al_2_O_3_·CaCl_2_·10H_2_O, FS) [34,35]. This process accelerates the consumption of Ca(OH)_2_ as well as Si- and Al- phases, generating additional compounds capable of enhancing the microstructure of the gel materials. Consequently, although CaCl_2_ does not directly participate the hydration reactivity of ISWs, it promotes the formation of hydration products and dissolution of ISWs, demonstrating potential as an effective chemical activator [34,35,36].

Given the high natural porosity and absence of fine particles in aeolian sand [4,5,6], the densification improvement induced by expansive agent incorporation may exhibit greater potential in augmenting the strength of ASIG. In existing studies, laboratory-synthesized MgO-based and CaO-based expansive agents have been extensively employed as additives in ultra-high-performance concrete (UHPC) to mitigate autogenous shrinkage [37,38,39,40,41]. However, the synthesis processes entailed relatively high production costs. Bentonite (BE), a natural clay extensively distributes in North, East, and Northwest China, is composed predominantly of montmorillonite. Due to pronounced water absorption, swelling capacity, and adsorption properties, it is often classified as an undesirable foundation material requiring treatment [42]. However, these intrinsic characteristics also endow it with potential as a natural expansive agent. Current research has demonstrated its application in preparing backfill materials or water-blocking agents in mines and oilfields engineering [42,43,44]. Additionally, desulfurized gypsum (DG) is an ISW generated through the reaction of limestone with SO_2_ in combustion flue gases from coal- or oil-fired power plants [45], containing CaSO_4_·2H_2_O that serves as an essential reactant for ettringite (3CaO·Al_2_O_3_·3CaSO_4_·32H_2_O, AFt) synthesis [46,47], thereby qualifying it as another potential expansive agent. Previous studies have used DG to develop ISWG, confirming its applicability. The effects of its dosage on the performance of geopolymer s and mechanisms have also been investigated [46,47,48,49].

In previous investigation [50], the efficacy of ISWG—formulated through synergistic integration of SS, GGBFS, phosphorus slag (PS), and CS—has been experimentally and comparatively validated through laboratory tests and literature benchmarking, demonstrating equivalent aeolian sand stabilization performance to OPC, and the optimal proportion of ISWs was determined using a dual-layer optimization strategy combining three chemical moduli theory with mixture design experiments. Compared with existing formulations determined through trial-and-error experiments [16,17,20], the primary oxide ratios of this geopolymer are more similar to those of OPC, resulting in lower risks in both application and durability. However, the findings also revealed the low activity of certain ISWs and potential for further strength enhancement.

Currently, studies on alkali-activated geopolymer stabilized soil primarily focus on clay or silty soils—types of problematic soils characterized by high natural water content and high clay fraction [16,18,20,23,24,26]. These soils possess inherent cohesion and sufficient pore fluid, allowing gels formed by geopolymers to disperse effectively and bind particles into an integral whole. In contrast, for aeolian sand, it’s extremely low moisture content [5,6,9] may pose significant challenges for the hydration of geopolymers. Thus, both the stabilization mechanism and effectiveness observed in clay soils cannot be directly extrapolated to aeolian sand.

On the other hand, current applications of expansive agents are mainly targeted at materials like UHPC, which contain well-graded aggregates whose pores are effectively filled by fine particles [37,38,39]. In these systems, the primary role of expansive agents is to mitigate shrinkage. However, for aeolian sand, its uniformly graded particle size distribution results in relatively large, unfilled pores between grains [7,9,11,50]. Whether this unique structure could allow expansive agents to provide enhanced strengthening effects remains a question that requires further clarification.

This study systematically investigated the effects of chemical activator and expansive agent dosage on 7, 14, and 28-day unconfined compressive strength (UCS) of desert aeolian sand stabilized by a quaternary ISWG prepared using GGBFS, SS, PS and CS. The selected chemical activators are NaOH, Ca(OH)_2_, and CaCl_2_, which represent three widely used major activation mechanism types: sodium-based strong alkali activators, calcium-based alkaline activators, and calcium-based salt activators, respectively. The chosen expansive agents are bentonite and desulfurized gypsum, representing natural clay mineral-based and industrial waste-based expansive agents, respectively. Optimal activators and expansive agents types with corresponding dosages for strength improvement were identified. Through phase evolution, microscopic morphology, and pores characterization, as well as pH variation, the enhancement mechanisms of chemical activators and expansive agents on ASIG have been elucidated. Compared to existing eco-friendly or bio-based stabilizers, the expansive agent-based approach developed in this study places greater emphasis on pore-filling efficacy and demands lower water content for effective soil stabilization. This mechanism is more precisely tailored to the distinct microstructure and pore characteristics of aeolian sand, thereby delivering a more effective stabilization solution.

## 2. Results and Discussion

### 2.1. Results of Material Characterization

**Figure 1:**
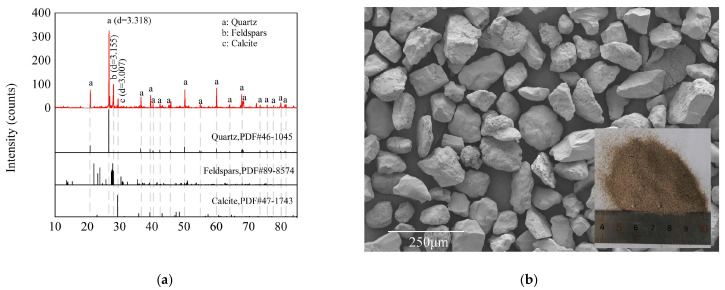
Phase composition and microscopic morphologies of aeolian sand: (**a**) XRD patterns reveal the mineral composition and (**b**) SEM Observation illustrate the microscopic morphology of particles.

**Figure 2:**
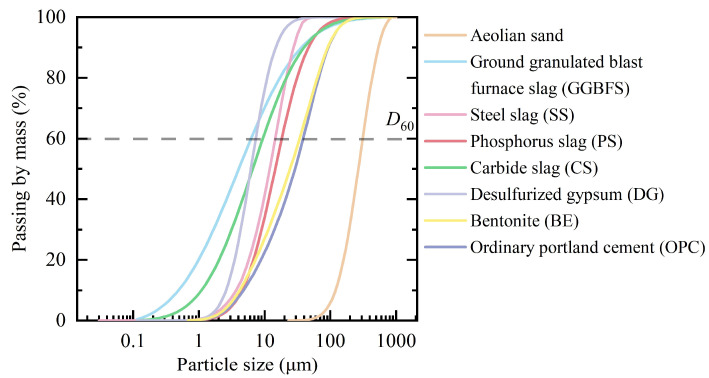
Particle size distributions of materials (*D*_60_ denotes the particle size for which 60% of the total mass of soil particles consists of particles smaller than this diameter).

XRD pattern and SEM observation of aeolian sand are shown in Figure 1. It can be identified that highly crystalline quartz constitutes the predominant mineral component in aeolian sand, accompanied by minor quantities of albite and microcline. The untreated sand particles exhibited smooth surfaces and high sphericity. Particle size distribution obtained by laser particle size analysis in Figure 2 classified aeolian sand as poorly graded medium sand, with *D*_60_ = 75 μm, uniformity coefficient *C*_u_ = 2.3, and curvature coefficient *C*_c_ = 2.5.

**Figure 3:**
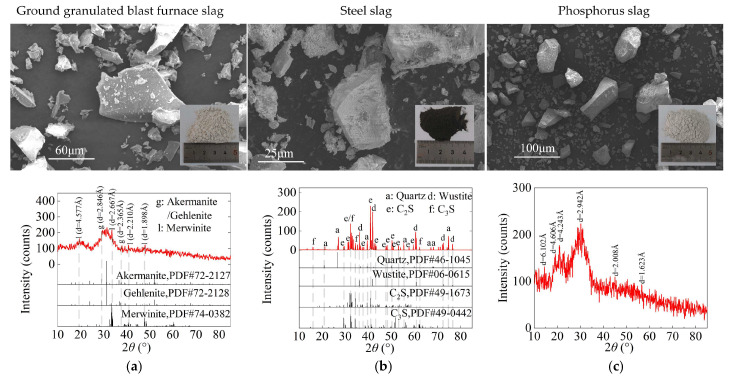

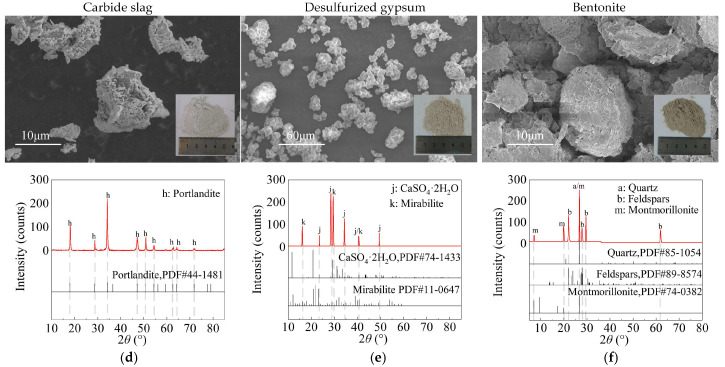
SEM observation and XRD pattern of ISWs and expansive agents: (**a**) Ground granulated blast furnace slag (GGBFS); (**b**) steel slag (SS); (**c**) phosphorus slag (PS); (**d**) carbide slag (CS); (**e**) desulfurized gypsum (DG); and (**f**) Bentonite (BE).

The macroscopic morphology, microstructural particle characteristics, and XRD patterns of ISWs are presented in Figure 3a–d. X-ray fluorescence (XRF) analysis determined the chemical compositions of ISWs in Table 1. The four ISWs were selected to achieve complementary chemical interactions: GGBFS and PS, characterized by their high content of Si/Al-phase oxides, low crystallinity, and high reactivity, are utilized as the primary sources of hydration reaction precursors for the formation of gel or mineral phases. While the main function of is to supplement Fe-oxides, which are absent in other components. CS specifically contributed CaO to enhance alkalinity and augment Ca^2+^ content within the system.

On the other hand, among the expansive agents used, the primary mineral component of desulfurized gypsum is dihydrate gypsum (CaSO_4_·2H_2_O), which serves as an essential precursor for the synthesis of ettringite. Its addition is therefore expected to promote the formation of ettringite. Characteristic peak of mirabilite (Na_2_SO_4_·10H_2_O) were also identified in the XRD pattern of desulfurized gypsum. It is a common impurity introduced during the production process. In contrast, the main mineral in bentonite is montmorillonite, a clay mineral characterized by a sandwich-type crystalline structure consisting of silica tetrahedral–alumina octahedral–silica tetrahedral layers. Exchangeable cations (such as Na^+^ or Ca^2+^) residing in the interlayer spaces can adsorb water molecules [51], leading to an increase in interlayer spacing and resulting in expansion behavior.

Particle size distributions of ISWs are also shown in Figure 2. It can be observed that the particle size distribution of the adopted materials is similar to that of cement, though the overall particle size is generally smaller than that of aeolian sand.

### 2.2. Effect of Additives on UCS of ASIG

**Figure 4:**
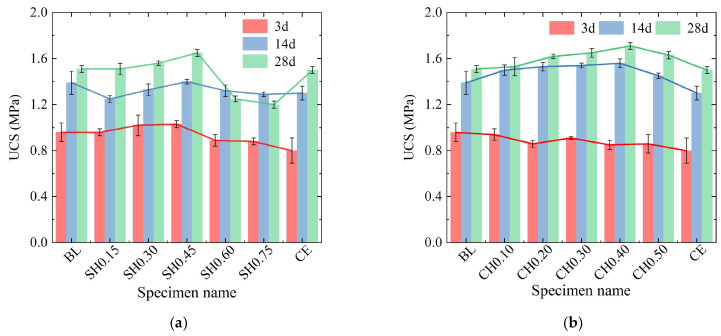

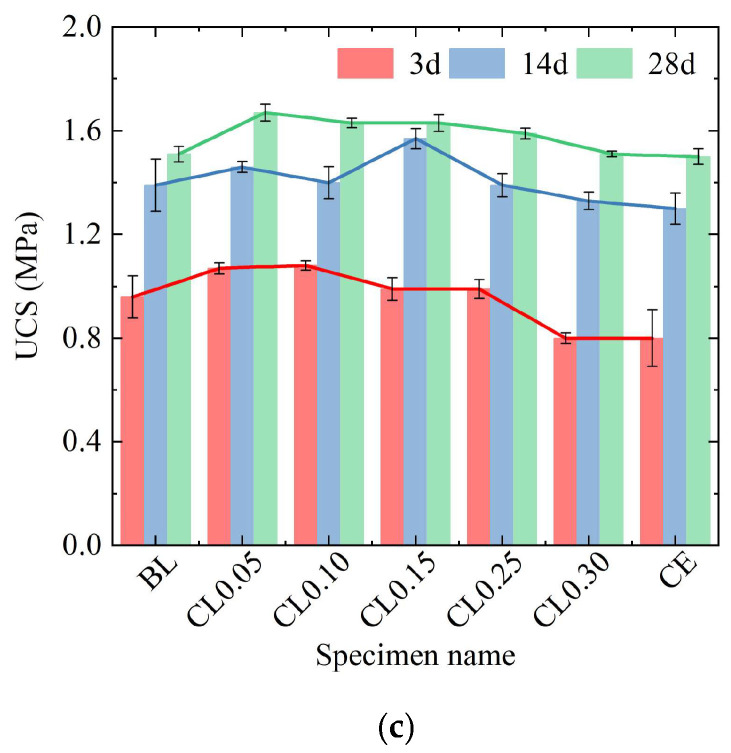
Effect of activators type and dosages on UCS of ASIG: (**a**) UCS increases with the low dosage but regression with the high dosage of NaOH; the UCS first increases and then decreases with the increase in the dosage of (**b**) Ca(OH)_2_ and (**c**) CaCl_2_, without showing regression.

Figure 4 illustrates the influence of three activator dosages on the UCS and strength development with curing age of ASIG, with comparative analysis against cement-stabilized aeolian sand at equivalent dosages. The results demonstrated that all three investigated activators exhibited discernible effects on the UCS of specimens. For NaOH, shown in Figure 4a, the UCS at all three curing ages exhibited an initial increase followed by a decrease with increment of concentration, reaching peak strength at 0.45 M. The error bars indicate that the difference was not statistically significant when the NaOH concentration was below 0.45 M but became significant above this threshold. The trend of UCS variation with NaOH dosage became more pronounced as the curing age increased, as evidenced by the UCS variation range increasing from 0.15 MPa at 3 days to 0.45 MPa at 28 days, and error analysis indicated that the influence of NaOH dosage became increasingly significant. Notably, exceeding the 0.45 M NaOH threshold induced strength regression, with concentrations of 0.60 M and 0.75 M resulting in UCS reductions of 0.07 MPa and 0.09 MPa, respectively. Additionally, ASIG incorporating NaOH demonstrated higher 3 days UCS compared to OPC-stabilized aeolian sand across all dosage levels. However, upon the curing time reaching day 28, this strength advantage persisted only in the ASIG specimen with concentration of NaOH being not more than 0.45 M.

Figure 4b illustrates the influence of Ca(OH)_2_ dosage on the UCS of ASIG. At 3 days of curing, the UCS exhibited minimal variation (range: 0.04 MPa) with no discernible correlation to Ca(OH)_2_ content. The variation in UCS with higher Ca(OH)_2_ dosage did not significantly exceed the standard deviation of the replicate measurements within each group, suggesting negligible early-age effects. As curing time extended to 14 and 28 days, the UCS range increased to 0.17 MPa and 0.20 MPa, respectively, revealing a gradual intensification of dosage dependency. Specifically, UCS initially increased with rising Ca(OH)_2_ concentration, peaking at 0.4 M, where strength improvements reached 12% and 13% compared to the control group BL at 14 and 28 days. Although error bars between some adjacent groups overlap, suggesting that minor variations in these localized ranges is unreliable, the overall trend remains robust and is less affected by error. Notably, no strength regression was observed at any dosage throughout the curing period. In contrast to cement-stabilized aeolian sand, Ca(OH)_2_-modified ASIG demonstrated consistently higher UCS values across all tested concentrations and ages, highlighting its enhanced and more steady activation potential relative to NaOH.

Figure 4c shows the effect of CaCl_2_ concentration on the UCS of ASIG at varying curing ages. UCS of specimens generally exhibited a trend of initial increase followed by a decrease with increasing concentration. At curing ages of 3 days, 14 days, and 28 days, the UCS ranges of the specimens were 0.12 MPa, 0.28 MPa, and 0.16 MPa, respectively. The peak UCS values consistently corresponded to concentration below 0.15 M. In contrast to NaOH, higher concentrations of CaCl_2_ did not induce strength regression in ASIG. At a 28-day curing age, the peak UCS of the specimen reached 1.67 MPa, exceeding the control groups BL and CE by 0.16 MPa.

**Figure 5:**
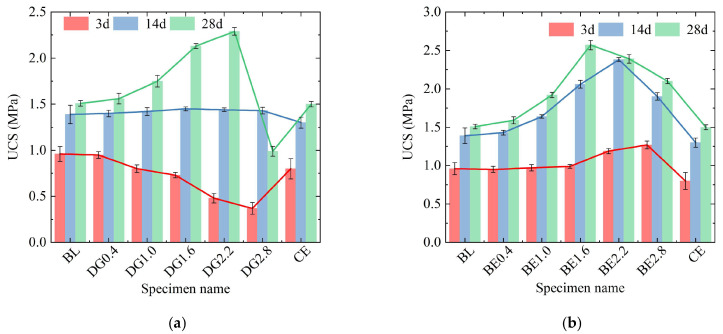
Effect of expansive agents type and dosages on UCS of ASIG: (**a**) The enhancement of UCS by the low dosage and the strength regression caused by the high dosage of desulfurized gypsum and (**b**) the most significant enhancement effect of bentonite on UCS.

Figure 5a,b, respectively, demonstrate effects of expansive agents DG and BE on ASIG strength. For DG in Figure 5a, specimens cured for 3 days exhibited a monotonic decline in UCS with increasing dosage, reaching a 61% reduction at 2.8% DG compared to control BL. Extended curing altered this trend: At the 14-day curing age, specimens with higher DG incorporation levels exhibited accelerated strength development, leading to minimal UCS variations among ASIG with different DG contents while marginally surpassing the control group. Upon reaching the 28-day curing age, specimens containing less than 2.8% DG had continued UCS enhancement, with values positively correlated with DG content, peaking at 2.29 MPa with a DG content of 2.2%. However, specimens incorporating 2.8% DG displayed significant strength reduction of 56.7% and even regression compared to their 14-day counterparts, and the substantial separation between the error bars confirms that this effect is statistically significant.

In contrast, BE-modified specimens in Figure 5b showed monotonic early-stage strength gains, with 2.8% BE yielding 1.27 MPa UCS at 3 days—58% higher than CE. The extension of curing age transformed the BE dosage–UCS relationship into a unimodal pattern. Specifically, specimens with 2.2% BE dosage achieved peak UCS values of 2.38 MPa at 14-day curing, exceeding the control group by 83%. This optimal dosage decreased to 1.6% at 28-day curing, corresponding to a UCS of 2.57 MPa that surpassed the control group by 70%. Moreover, after the curing age reached 14 days, the error bars of all specimens showed no overlap, indicating that the UCS was significantly influenced by the dosage of BE. It is noteworthy that BE-modified specimens exhibited higher UCS values compared to those adjusted with other additives across all curing ages, highlighting BE’s comparatively superior strengthening capability for ASIG.

A comparative analysis of strength was conducted with existing studies utilizing similar ISWs-based geopolymers for stabilizing problematic soils. Wu et al. [26] stabilized marine soft clay using SS and fly ash, achieving a maximum UCS of 1.2 MPa, which is 53% lower than the peak strength obtained in this study for ASIG. In another study by the same group [52], where alkali-activated SS was used to stabilize expansive soil, the reported maximum strength was 1.68 MPa, 34% lower than that of ASIG. Luo et al. [53] employed a GGBFS-based geopolymer to stabilize riverine soft clay; with a binder content of 18%, the stabilized soil achieved a strength of 1.72 MPa, 33% lower than the strength reported herein for aeolian sand. In a further example, Wu et al. [54] utilized a fly ash/slag-based geopolymer to stabilize soft clay, reaching a strength of 2.0 MPa at 22% binder content, which remains 5% lower than the strength of ASIG. These comparisons demonstrate that the stabilized soil enhanced with BE in this study exhibits a distinct strength advantage.

### 2.3. Evolution of Reaction Products

**Figure 6:**
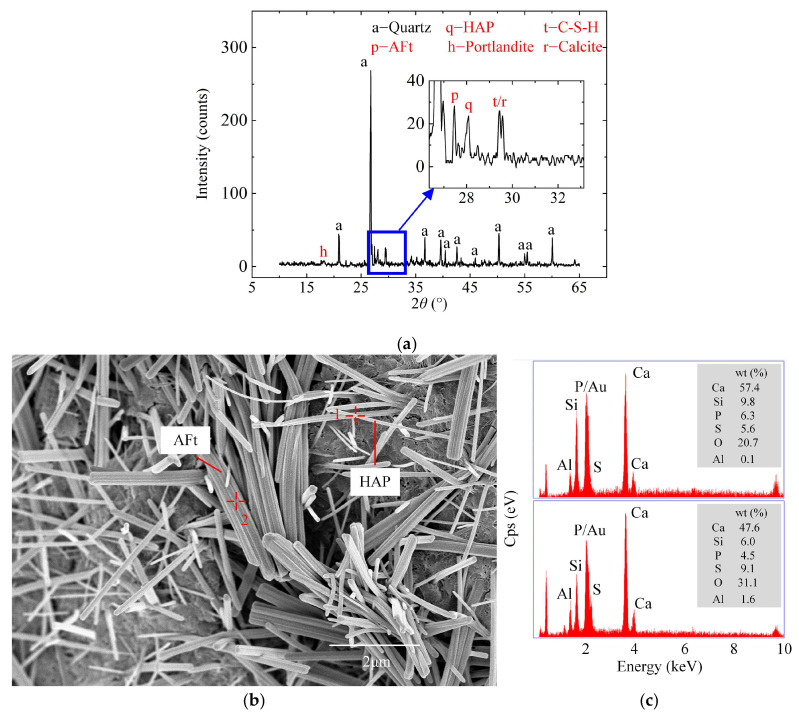
Phase analysis of specimens in control group BL: (**a**) The XRD patterns show characteristic peaks of hydration products such as C(-A)-S-H, AFt, portlandite, calcite, and HAP; (**b**) Rod-like HAP crystals are observed in the SEM images; and (**c**) EDS confirms the presence of P element in the rod-like crystals.

The effects of different types and dosages of additives on the phase composition of ASIG were investigated through XRD, TG analyses to elucidate strength variations. Figure 6a presents the XRD pattern of the control group (BL) after 28 days of curing. Since SiO_2_ present in aeolian sand primarily exists in the form of highly crystalline quartz, which is widely regarded as being scarcely reactive in hydration processes, the prominent quartz diffraction peaks observed in the XRD patterns were not emphasized in the phase analysis. The results demonstrate that the hydration products of ISWG primarily consist of C-S-H gel (2*θ* = 29.5°, *d* = 3.020 Å), AFt (2*θ* = 27.5°, *d* = 3.240 Å), calcite (2*θ* = 29.4°, *d* = 3.035 Å), and minor portlandite (2*θ* = 18.0°, *d* = 4.922 Å), which were analogous to OPC. However, a distinct characteristic peak of hydroxyapatite (Ca_10_(OH)_2_(PO_4_)_6_, HAP) was observed at 2*θ* = 28.1° (*d* = 3.170 Å). HPA is a mineral with hexagonal crystal system and its needle-like or rod-like morphology is similar to that of AFt. Formation of this phase may be attributed to PO_4_^3−^ released by dissolution of PS reacting with Ca^2+^ and OH^−^ derived from other ISWs, as described in Equation (1) [55]. Such reaction was commonly employed for phosphorus precipitation and removal from industrial wastewater using ISWs [56,57].

(1) $$10{\mathrm{Ca}}^{2+}+6{\mathrm{PO}}_{4}^{3-}+2{\mathrm{OH}}^{-}\to {\mathrm{Ca}}_{10}{\left(\mathrm{OH}\right)}_{2}{{(\mathrm{PO}}_{4})}_{6}↓$$

To further verify the presence of HAP, the micro-morphology of the specimen from the control group BL that had been cured for 28 days was observed using SEM and shown in Figure 6b. A large number of rod-shaped crystals with different aspect ratios were found. EDS point scanning analyses were, respectively, carried out on the thicker and thinner crystals, and the obtained spectra are shown in Figure 6c. Besides the sputtered gold, the results indicated the existence of six elements: O, Al, Si, Ca, S, and P. Due to the application of a relatively high accelerating voltage, the electron beam penetrated the crystals and reached the underlying phase, resulting in the appearance of Si and an excess of Ca. Such errors are common, and the types of minerals can be determined according to the ratios of elements. Point 1 was located on the thinner crystal. It can be seen that the content of P was higher than that of S, and the amount of Al was extremely low. In contrast, the spectrum of point 2 located on the thicker crystal showed a relatively high content of S and Al, along with a lower content of P. These ratios of elements, respectively, correspond to the elemental composition characteristics of HAP and AFt. In addition, the elongated rod-shaped crystal was also consistent with the morphology of HAP synthesized in alkaline condition observed in the literature [58,59,60]. The XRD results and the presence of P in EDS is in any case a clear indication about the occurrence of HAP.

#### 2.3.1. XRD Pattern

**Figure 7:**
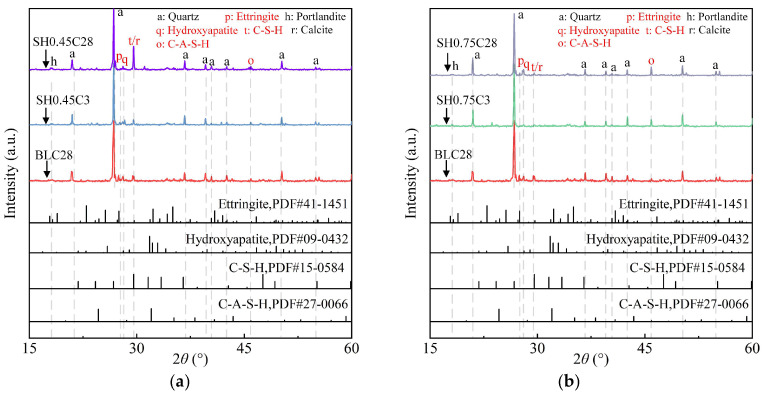

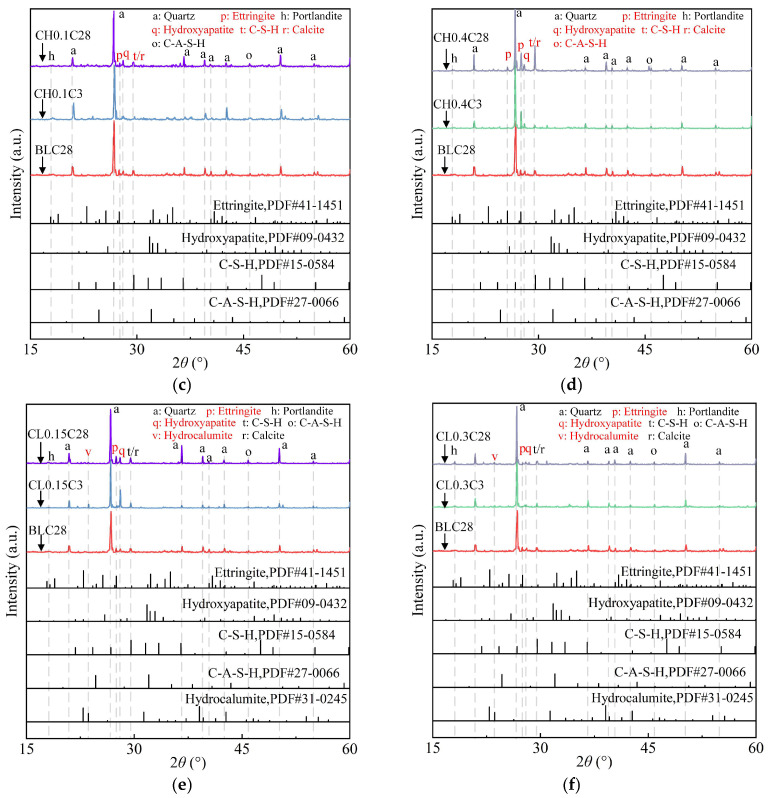
XRD pattern of ASIG with different activators type and dosages: (**a**) The low dosage of NaOH leads to an increase in the diffraction peaks of C-S-H/calcite, while (**b**) the high dosage causes a decrease in the product peaks; (**c**,**d**) The incorporation of Ca(OH)_2_ enhances the intensity of the diffraction peaks of AFt and HAP; (**e**) The introduction of CaCl_2_ results in the appearance of characteristic peaks of hydrocalumite, and (**f**) the low dosage promotes the synthesis of AFt.

Figure 7 presents the XRD patterns of ASIG incorporating different types and dosages of activators. The promotion or inhibition of hydration products by the additives can be semi-quantitatively analyzed based on the relative intensities of the diffraction peaks in the pattern. Therefore, arbitrary units (a.u.) are used as the unit for peak intensity.

For NaOH-modified specimens, a concentration of 0.45 M led to early observation of C-S-H/calcite and HAP peaks in 3 days (SH0.45C3) and enhancement after 28 days (SH0.45C28), while the intensity of peak corresponding AFt remained low. Increasing concentration to 0.78 M resulted in predominant C-A-S-H (2*θ* = 45.9, *d* = 1.974 Å) formation at 3 days (SH0.75C3), followed by HAP peak intensification at 28 days with minimal changes in other phases (SH0.75C28). The increased C-A-S-H formation may be attributed to accelerated dissolution of ISWs to release Al^3+^, which intercalated into the interlayer structure of C-S-H, facilitating its transformation into C-A-S-H [61,62].

In Ca(OH)_2_-modified systems, low dosage specimens showed only faint C(-A)-S-H peaks at 3 days (CH0.1C3) with minimal AFt detection. Higher Ca(OH)_2_ dosage promoted early AFt and HAP formation at 3 days (CH0.4C3). For specimen with a curing age of 28 days (CH0.4C28), distinct characteristic peaks of C-S-H/calcite were observed. Additionally, a new characteristic peak of AFt appeared at 2*θ* = 25.6° (*d* = 3.475 Å), accompanied by enhancement in the peak intensity of HAP. The enhanced AFt synthesis may originate from Ca^2+^ concentration surpassing nucleation thresholds [63] due to introduction of Ca(OH)_2_ (explaining why equivalent NaOH addition failed to promote AFt synthesis). It is also noteworthy that the characteristic peak of portlandite at 2*θ* = 18.0° disappeared. This is presumably due to the consumption of a substantial amount of Ca(OH)_2_ by the hydration reactions occurring in the system, as well as its partial carbonation into calcite, which corresponds to the observed increase in the intensity of the characteristic peaks of calcite.

For CaCl_2_-modified specimens, characteristic peak of the newly formed hydration products, hydrocalumite, was identified at 2*θ* = 22.5° (*d* = 3.940 Å) and 23.0° (*d* = 3.850 Å) in all patterns due to Cl^−^ introduction. The intensity of AFt peaks was initially low (CL0.15C3) because of the preferential reaction of Al(OH)_4_^−^ with Cl^−^ to form hydrocalumite, while the synthesis of HAP was promoted due to the supplementation of Ca^2+^, reflected in its enhanced characteristic peak. After 28 days of curing (CL0.15C28), the peak intensity of AFt was enhanced, which is likely attributed to the depletion of Cl^−^ in the system, which allowed the remaining Al(OH)_4_^−^ ions to participate in the formation of AFt. However, a higher CaCl_2_ concentration (0.3 M) reduced the diffraction peak intensities of both AFt and HAP.

**Figure 8:**
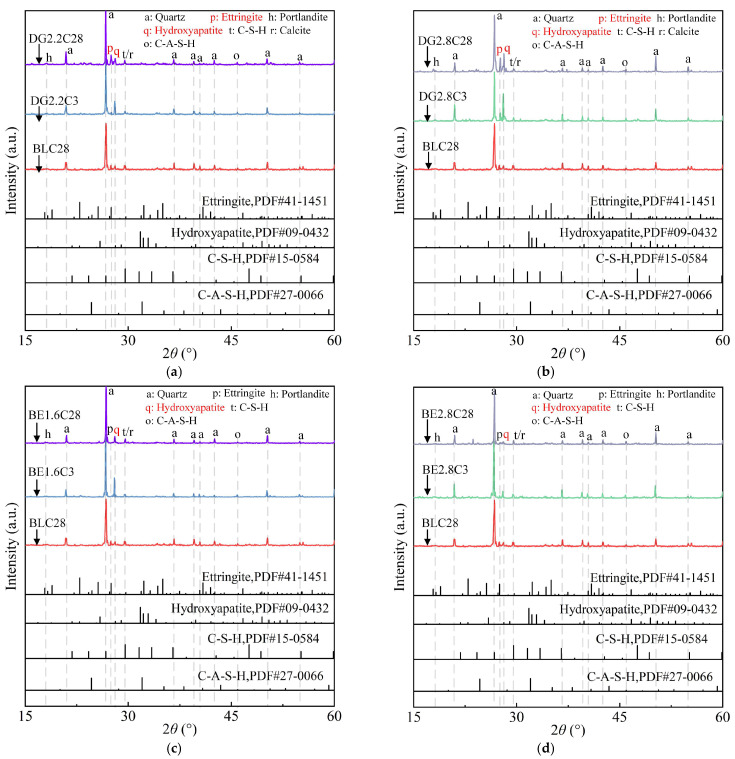
XRD pattern of ASIG with different expansive agents type and dosages: (**a**) The incorporation of DG leads to an enhancement in the diffraction peak of ettringite; and (**b**) the enhancement becomes more pronounced at higher dosage levels; (**c**) the incorporation of a low dosage of BE enhances the diffraction peak of HAP during the early curing age; and (**d**) the effect of BE becomes less discernible in the diffraction patterns at higher dosage levels.

Figure 8a presents the XRD patterns of ASIG modified with 2.2% and 2.8% DG, corresponding to specimens with the highest 28-day UCS and that exhibiting strength regression, respectively. In DG-modified systems, distinct HAP peaks emerged after 3 days of curing (DG2.2C3 and DG2.8C3), alongside enhanced portlandite signals due to CaO hydration in DG. By 28 days (DG2.2C28 and DG2.8C28), AFt peak intensity increased significantly, with higher DG content yielding more prominent AFt peaks. This enhancement can be attributed to the dual role of DG, which supplies both the necessary Ca^2+^ and SO_4_^2−^ ions for AFt formation. Furthermore, the hydration of CaO elevates the alkalinity of the system, creating a more favorable environment for AFt synthesis. It is noteworthy, however, that although both DG and Ca(OH)_2_ contributed to the promotion of AFt formation, the XRD pattern of the DG-modified specimen did not exhibit an additional characteristic peak of AFt at 2*θ* = 25.6° (*d* = 3.475 Å). This suggests a potential difference in the mineralogical morphology of AFt formed in the two systems.

Figure 8b shows the impact of varying BE dosages: specimens with 1.6% BE exhibited strong HAP and resolvable C-S-H peaks at 3 days (BE1.6C3), indicating that the addition of BE enhanced the crystallinity or increased the yield of HAP. Conversely, 2.8% BE incorporation weakened HAP signals at 3 days (BE2.8C3). After 28 days, HAP diffraction intensity in 1.6% BE specimens diminished. While no evidence suggests HAP dissolution in the alkaline system, this attenuation may stem from either morphological transformation of HAP crystals or physical shielding by amorphous gel phases, which attenuate XRD signals. However, further microstructural and phases analyses are necessary to validate this hypothesis.

#### 2.3.2. TG/DTG

**Figure 9:**
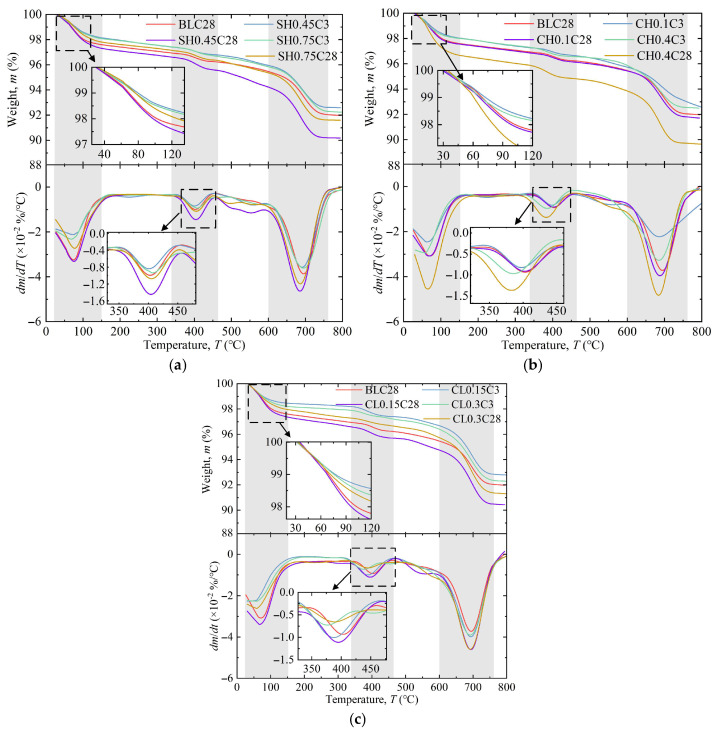
TG/DTG curve of ASIG with different activators type and dosages: (**a**) A moderate dosage of NaOH leads to an increase in mass loss; (**b**) the introduction of Ca(OH)_2_ causes a left shift in the DTG peak in the second stage; and (**c**) the incorporation of CaCl_2_ reduces the mass loss in the second stage.

Figure 9 presents the TG/DTG curves of specimens modified with activators, providing complementary insights into the relative content of hydration products. The mass loss of ASIG primarily occurs in three distinct stages: Stage 1 (40–150 °C) corresponding to dehydration of C-(A)-S-H gel and AFt; Stage 2 (350–450 °C) associated with dehydroxylation of Ca(OH)_2_ and HAP; and Stage 3 (600–750 °C) representing calcite or other mineral decomposition [26].

At 3 days of curing, ASIG incorporating 0.75 M NaOH displayed slightly elevated mass loss (about 0.1%) compared to that with 0.45 M NaOH, with more pronounced differences in Stages 2 and 3. This pattern, however, reversed at 28 days: the losses in Stage 1 and Stage 2 of specimen SH0.45C28 were 13.8% higher and 35.3% higher than those of the control group, respectively. In contrast, the losses for specimen SH0.75C28 were 8.9% lower and 3.3% lower than the control group, implying impaired hydration product synthesis at higher alkalinity. The addition of 0.4 M Ca(OH)_2_ enabled specimens to achieve Stage 2 mass loss comparable to BLC28 after only 3 days curing. Specimens CH0.4C28 demonstrated 0.75% increased mass loss in the first stage, exceeding the enhancement induced by optimal NaOH concentration, consistent with intensified peak intensities in XRD patterns. Notably, Ca(OH)_2_-modified specimens exhibited leftward peak shifts in Stage 2 of DTG curves, likely due to increased HAP formation whose dehydroxylation occurs at lower temperatures than Ca(OH)_2_ [64]. Addition of 0.15 M CaCl_2_ also promoted AFt and HAP formation, but its elevation to 0.3 M significantly reduced Ca(OH)_2_ content, suggesting that the synthesis of hydrocalumite due to excessive Cl^−^ introduction consumed more OH^−^ and thereby accelerated Ca(OH)_2_ decomposition.

**Figure 10:**
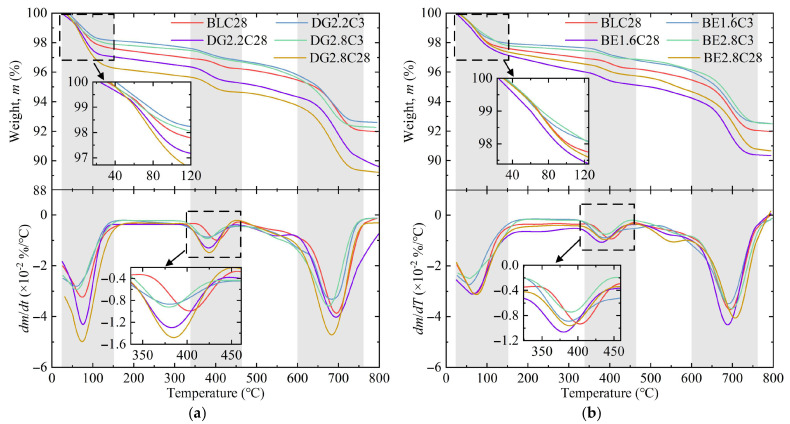
TG/DTG curve of ASIG with different expansive agents type and dosages: (**a**) The incorporation of desulfurized gypsum increases the mass loss in the first two stages and causes a left shift in the peak in the second stage and (**b**) the incorporation of bentonite has little effect on the TG/DTG curves.

The thermogravimetric results of ASIG modified with DG and BE are illustrated in Figure 10. The CaSO_4_·2H_2_O present in DG provided abundant precursors for AFt formation [65], which led to a significant increase in mass loss during the first stage for DG-modified specimens curing for 28 days (DG2.2C28 and DG2.8C28 exhibited 24.3% and 134.0% higher than control group). This observation, combined with the intensified AFt diffraction peaks in the XRD patterns, indicates substantial AFt generation within the system, with the quantity increasing proportionally to DG dosage. However, this trend was not prominent in 3-day-cured specimens, likely attributable to the delayed release of CaSO_4_·2H_2_O caused by the relatively low reactivity of DG. Furthermore, enhanced mass loss in the second stage and leftward-shifted/broadened DTG peaks were observed, suggesting that the hydration of CaO in DG generated additional Ca(OH)_2_, resulting in elevated alkalinity and Ca^2+^ concentration, which promoted HAP synthesis, consistent with XRD analysis. In contrast, BE-modified specimens exhibited increased HAP production at 3 days of curing but showed no significant difference in first-stage mass loss. This implies that the Si- and Al-phase oxides in BE exhibited limited capacity to serve as precursors for hydration reactions.

### 2.4. Characteristic of Microscopic Morphology and Structure

#### 2.4.1. Morphology of Hydration Products

**Figure 11:**
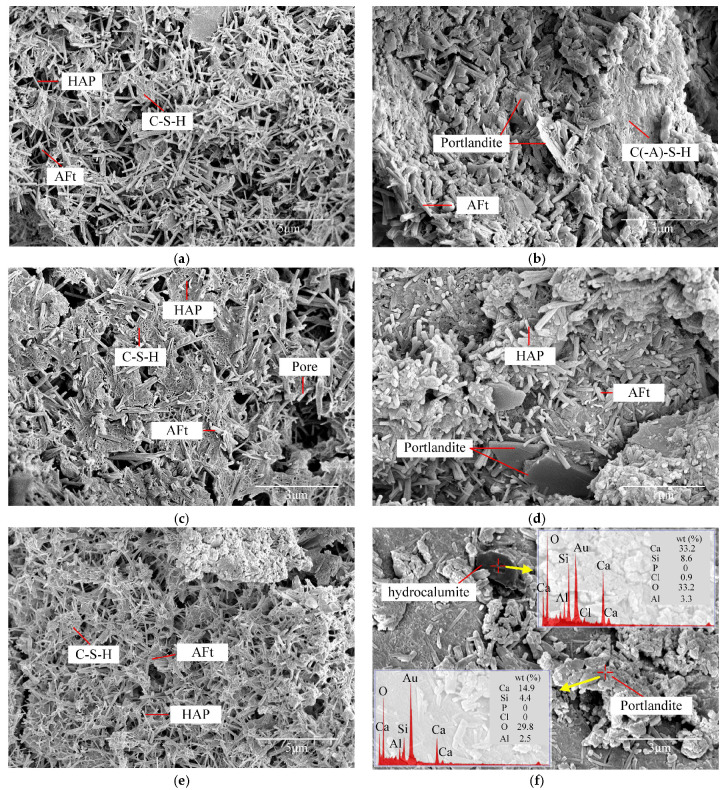

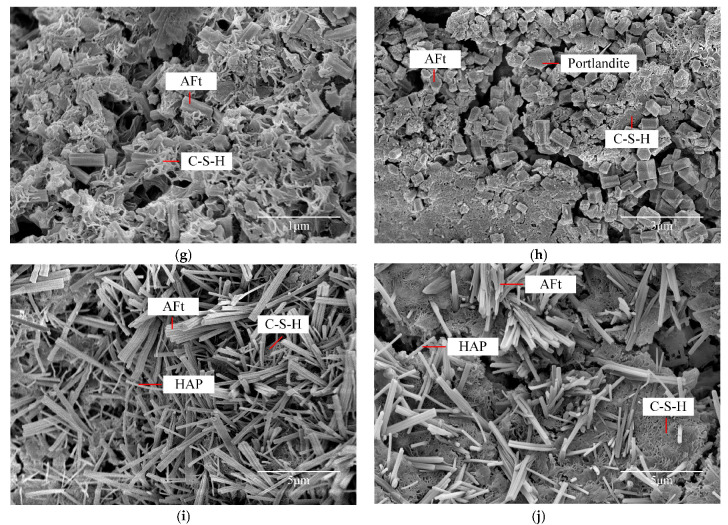
Hydration products morphologies of specimens: (**a**) The porous microstructure of SH0.45C3 and (**b**) the reaction products of SH0.45C28 are more compact; the plate-like portlandite appearing from (**c**) CH0.4C3 to (**d**) CH0.4C28; the hydrocalumite crystals generated from (**e**) CL0.15C3 to (**f**) CL0.15C28; the short columnar AFt crystals appearing due to the incorporation of DG in (**g**) DG2.2C3 and (**h**) DG2.2C28; (**i**) a relatively large number of HAP crystals in BE1.6C3; and (**j**) being covered by gels in BE1.6C28.

Figure 11 illustrates variations in hydration products morphologies encapsulating sand particle of ASIG modified with different additives. C-(A)-S-H gel, AFt, and HAP were ubiquitously identified across all specimens. For alkali-activated specimens, hydration products exhibited relatively loose structures at 3 days, transitioning to denser configurations by 28 days with observable formation of plate-like portlandite, consistent with XRD and TG analyses. Specimens modified with CaCl_2_ demonstrated hydrocalumite formation at 28 days, which can be confirmed through EDS detection of elevated chlorine content. In DG-modified specimens, AFt crystals displayed smaller aspect ratios, potentially attributable to nucleation alterations induced by supplementary CaSO_4_·2H_2_O introduction. Finally, BE-modified ASIG exhibited substantial HAP crystallization at 3 days of curing, with gel formation being virtually undetectable, corresponding to the high-intensity diffraction peaks in XRD patterns. This phenomenon may attribute to the montmorillonite in BE, as its crystalline structure may exert a beneficial effect on the growth of HAP. However, the underlying mechanisms remain unclear. Following 28 days of curing, a marked proliferation of gel was observed, partially obscuring the crystals, which may account for the reduction in diffraction peak intensity.

#### 2.4.2. Microstructures and Pore

**Figure 12:**
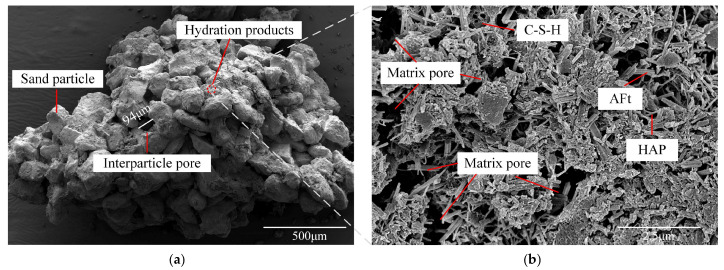
Morphology of a particle cluster of ASIG: (**a**) Micron-scale interparticle pores observed at low magnification and (**b**) nanoscale matrix pores at high magnification.

The microstructural characteristics of ASIG observed via SEM on fragments extracted from fractured specimen were shown in Figure 12. At low magnification, it can be observed that the aeolian sand particles were enveloped by hydration products of ISWs, forming interparticle connections that bind individual particles, which was considered as the primary source of ASIG strength. However, due to the relatively large and uniform particle size of aeolian sand, the interstitial spaces between particles formed pores with diameters approximately 94 μm. These pore dimensions exceed the diffusion range of hydration products, rendering them difficult to be filled by either hydration products or finer particles, consequently creating vulnerable regions within the ASIG. Therefore, the efficacy of interparticle pore filling emerges as a critical determinant for further enhancing the mechanical strength of ASIG. Higher-magnification examination of particle surfaces shown in Figure 12b reveals hydration products whose restricted quantity and varying crystallinity generated matrix pores. The areal fraction of pores within the matrix was postulated to serve as a quantitative metric for hydration product densification, dictated by the speciation and productivity of hydration phases. It emerged as a direct determinant of interparticle bond integrity and thereby affected the macroscopic strength of ASIG.

**Figure 13:**
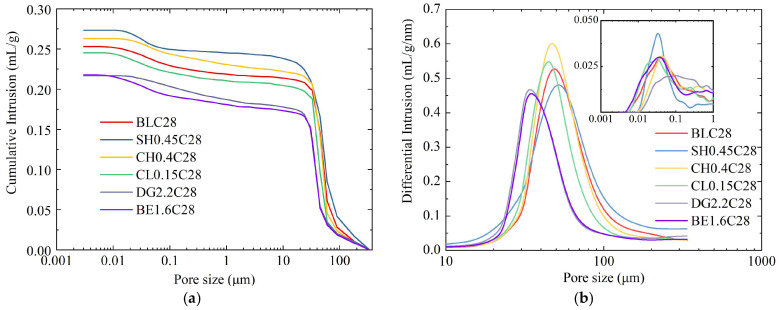
Pore size distributions of ASIG modified with different admixtures determined by MIP: (**a**) Pore size vs. cumulative mercury intrusion curve; (**b**) pore size vs. differential mercury intrusion curve.

The results of MIP are displayed in Figure 13. Specimens exhibiting the highest 28-day UCS from each group were selected to evaluate the effect of additives on pore size distribution in ASIG. As illustrated in the cumulative mercury intrusion-pore size curves shown in Figure 13a, all specimens demonstrated rapid mercury intrusion increment within the 10–100 μm pore size range, resulting from mercury penetration into larger interparticle pores. A secondary ascending trend emerged in the 0.01–0.1 μm range, corresponding to high-pressure intrusion into finer interparticle pores and matrix pores. Compared with the control group (BLC28), expansive agents-modified specimens (DG2.2C28 and BE1.6C28) exhibited reduction in total mercury intrusion volume, with more pronounced reduction observed in pores exceeding 40 μm, which predominantly represent interparticle pores. Conversely, alkali-activated specimens (SH0.45C28 and CH0.4C28) displayed higher intrusion volumes.

Additionally, the differential intrusion curves in Figure 13b revealed that the peak pore size of unmodified ASIG corresponds to approximately 45 μm, indicating that pores of this size dominate ASIG. The characteristic can be similarly observed in alkali-activated ASIG. In contrast, DG and BE modified specimens exhibited distinct leftward shifts in their peaks accompanied by reduced peak values. Specifically, the peak pore size of specimens modified with DG and BE decreased by approximately 30% compared to the control group, demonstrating a significant reduction in large interparticle pores. In addition, the sample modified by NaOH displayed a narrower and higher peak between 10 nm and 100 nm compared to the control group, suggesting increased concentration in the size distribution of finer pores. For CaCl_2_-modified ASIG (CL0.15C28), the large pore characteristics remained essentially identical to those of the control group, while the small pore peak showed a leftward shift.

**Figure 14:**
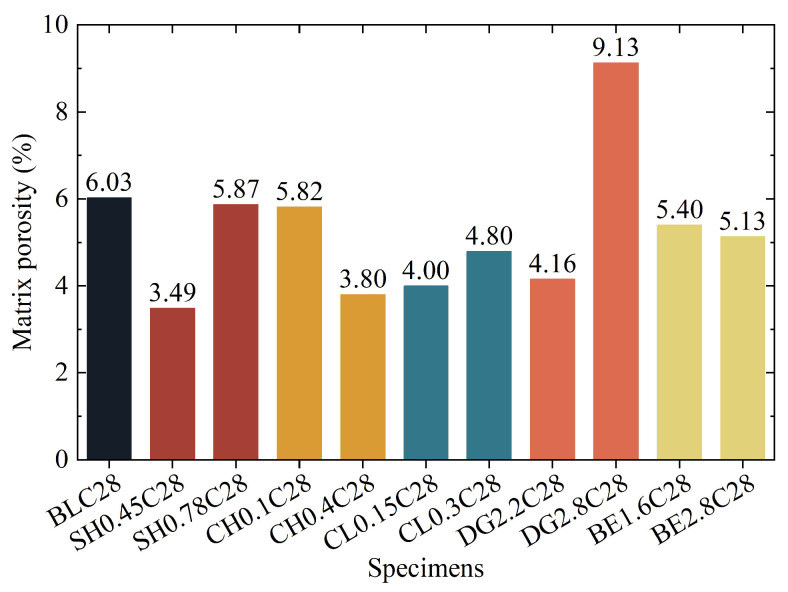
Matrix porosity of ASIG modified with different admixtures determined by SEM image-based identification methodology.

The SEM images utilized for pore identification are comprehensively compiled in Appendix A. Image-derived matrix porosity of selected specimens is presented in Figure 14. The 0.45 M NaOH-modified specimen exhibited a substantial reduction of 42% in matrix porosity compared to the control group (BLC28), indicating densification of hydration products, which aligns with the increased hydration product content observed in phase analysis and the enhanced macroscopic strength. However, when the concentration increased to 0.75 M, the matrix porosity rebounded to levels comparable to the control group due to reduced hydration product formation. In contrast, Ca(OH)_2_-modified ASIG demonstrated an inverse trend, where higher concentrations progressively decreased porosity. This phenomenon was attributed to the optimized packing efficiency of portlandite and AFt crystals in pore filling, as corroborated by phase analysis and microscopic morphology characterization. The optimal CaCl_2_ dosage similarly reduced matrix porosity, although distinct from alkali activators, the pore-filling crystals primarily consisted of hydrocalumite, AFt, and HAP. Excessive CaCl_2_ dosage diminished crystal quantities, resulting in renewed porosity increase. Incorporation of 2.2% DG significantly reduced matrix porosity by 31% through AFt proliferation, while overdosing (3.5%) drastically increased porosity to 9.13%, suggesting the destruction of the matrix. Comparatively, BE incorporation only marginally reduced matrix porosity overall.

### 2.5. Enhancement Mechanism of Chemical Activators

**Figure 15:**
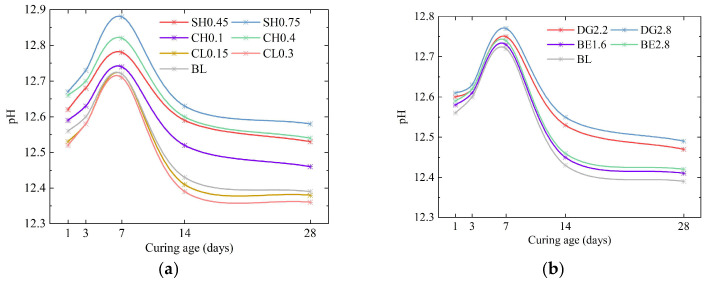
Variations in pH of ASIG modified with different additives with curing age: (**a**) The incorporation of higher concentrations of alkali activators may have elevated the system’s pH beyond the optimal threshold for hydration product formation; and (**b**) the addition of expansive agents exerts minimal influence on the pH of the system.

Comparative analyses of macroscopic strength reveal that the incorporation of alkaline activators (NaOH and Ca(OH)_2_) resulted in a maximum increase of only 13% in 28d UCS of ASIG and excessive dosages even led to reduced strength. This trend deviates from previous studies on ISWs-stabilized clays, where appropriate alkaline activators dosages were shown to exponentially enhance the strength of stabilized soils [66,67,68]. As well known, the primary purpose of adding alkaline activators is to increase the alkalinity of the system, thereby accelerating the release of precursors for hydration reactions from ISWs. Therefore, to explain the unique behavior observed in ASIG, pH of selected specimens at different curing ages were tested, as shown in Figure 15.

The pH of control group BL reached 12.56 at 1 day of curing, comparable to values reported for cement-stabilized clays in the literature [69,70,71]. As curing progresses, it exceeded 12.70 by 7 days and subsequently declined. Considering the water addition during measurement, pH of the ASIG pore solution may be higher than measured values. The initially high alkalinity of the specimens is attributed to the incorporation of substantial CS mainly containing CaO, which generate Ca(OH)_2_ after hydration. In other words, CS itself, as a component of the ISWG, inherently acts as an alkaline activator [72,73,74]. The addition of NaOH and Ca(OH)_2_ further enhances the alkalinity of the system, while also rendering it more prone to exceeding the upper limit suitable for the synthesis of hydration products. At moderate dosages, the increased OH^−^ concentration indeed accelerates the dissolution of ISWs and promotes the formation of hydration products, which can be corroborated by enhanced peak intensities in XRD patterns, increased mass loss in TG analysis and reduced matrix porosity. Conversely, excessive alkaline activator dosages introduce an overabundance of OH^−^ ions, which inhibit the synthesis of hydration products or alter the crystallization state of gels [75,76,77], resulting in a looser gel matrix that negatively impacts the strength of ASIG. Notably, test results indicate that Ca(OH)_2_-modified specimens exhibit superior strength performance compared to those modified by NaOH. This enhancement may originate from the relatively lower solubility of Ca(OH)_2_, which induces a more gradual pH increase in the system, while the additional Ca^2+^ facilitate the synthesis of HAP and AFt. Additionally, although the experimental characterization in this study did not provide direct evidence for substitution of C(-A)-S-H by sodium aluminosilicate hydrate (N-A-S-H) in NaOH- modified ASIG [78,79], such a phase transformation might contribute to strength limitations of the specimens.

**Figure 16:**
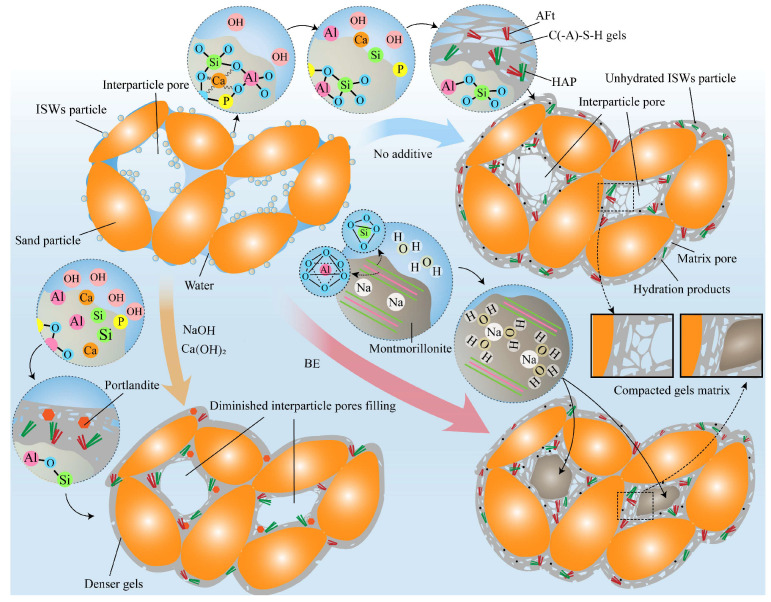
Enhancement mechanisms of alkaline activators and BE on ASIG.

Figure 16 provides an additional explanation. The stabilized aeolian sand, characterized by large interparticle pores and low moisture content, initially adsorbs water and fine ISWs particles around sand particles via capillary forces [80]. After curing, hydration products are formed from ISWs partially encapsulated the sand particles and create connections between them. However, limited dissolution of ISWs without activators release low concentrations of HSiO_3_^−^, Al(OH)_4_^−^, and PO_4_^3−^, which diffuse extensively, forming loose gels through delayed hydration reactions but fail to fill the interparticle pores. Optimal alkaline activation accelerates ISWs dissolution, generating localized high ion concentrations that rapidly form dense hydration products near particles surfaces (which has been more comprehensively characterized in the literature [81,82]). For stabilized soils with smaller particle sizes and lower porosity, the reduced formation range of hydration products induced by alkali activation can still adequately fill interparticle pores, and their densification contributes more directly to strength enhancement [83,84]. In contrast, for stabilized aeolian sand, this phenomenon further diminishes the extent of interparticle pores filling, which explains the increased mercury intrusion in MIP and ultimately limited strength development.

The incorporation of CaCl_2_ influences ASIG through distinct mechanism: First, it introduces more free Ca^2+^ ions compared to Ca(OH)_2_ due to relatively high solubility, thereby accelerating the synthesis of HAP and AFt; Second, Cl^−^ reacts with AlO_4_^−^ to form lamellar hydrocalumite crystals, which exert a pore-filling effect analogous to that of portlandite. This reaction enhances the dissolution of ISWs, thereby increasing the yield of hydration products [85]. Consequently, pore structure analysis reveals that CaCl_2_-modified samples exhibited a slight leftward shift in the interparticle pore size distribution peak and reduced matrix porosity. However, excessive Cl^−^ promote the conversion of AFt to hydrocalumite with consuming additional OH^−^ (evidenced by the diminished mass loss of Ca(OH)_2_ in the TG/DTG curves), which adversely impacts the synthesis of C(-A)-S-H gel and HAP, resulting in a porous matrix.

### 2.6. Enhancement Mechanism of Expensive Agents

The swelling behavior of BE primarily originate from montmorillonite, a mineral whose unit cell is widely recognized to exhibit a sandwich-like structure—two silica tetrahedral layers enclosing one alumina octahedral layer. Cations reside between adjacent unit cells [51]. Upon contact with water in the system, these cations undergo hydration by absorbing water molecules, a process that increases the interlayer spacing of the unit cells, manifesting as montmorillonite expansion. The swelling mechanism endows BE with dual roles in ASIG as shown in Figure 16: (1) Effectively fills interparticle pores, providing robust skeletal support, which can be evidenced by the significant reduction in large pores observed in MIP results; (2) Exerts compressive forces on the gel matrix encapsulating particles, leading to increased matrix density, as reflected by a slight decrease in matrix porosity. The presence of large interparticle pores is a distinctive feature of stabilized aeolian sand compared to stabilized fine-particle soils such as clays or silt. BE’s ability to fill these interparticle pores addresses the inherent limitation of ISWs-based geopolymers, which primarily serve to bind sand particles. Consequently, BE demonstrates a more pronounced macroscopic strengthening effect than alkaline activators, establishing it as a superior external additive for ASIG enhancement. However, this mechanism also implies that excessive BE incorporation may induce overexpansion, thereby subjecting the gel matrix connecting particles to tensile stresses or even fracturing it. The process simultaneously absorbs more water from the system and blocks capillary pores, hindering the dissolution of ISWs and the synthesis of hydration products [86,87]. The combined effect represents a plausible explanation for the observed strength reduction in ASIG when the BE dosage exceeds the optimal level.

As another expansive agent, DG influences ASIG through mechanisms distinct from those of BE. Phase analyses reveal that the substantial incorporation of CaSO_4_·2H_2_O elevates the production of AFt and HAP, resulting in enhanced diffraction peak intensities. SEM imaging further demonstrates that AFt morphology transitions from elongated rods to shorter prisms. This morphological shift likely enhances their pore-filling capacity within the gel matrix and increases the overall volume of hydration products, enabling coverage of a greater proportion of interparticle pores [88]. Additionally, Ca(OH)_2_ produced by hydration of CaO in DG increases pH of the system, thereby enhancing the dissolution of ISWs and promoting the synthesis of HAP. Consequently, synchronized reductions in interparticle and matrix porosity have been observed, which cause the improvement of UCS. However, excessive DG incorporation significantly increases matrix porosity. The amplified mass loss between 60 °C and 140 °C in TGA coupled with further enhancement of XRD diffraction peak intensities suggests the formation of a larger population of short-prismatic AFt crystals. These crystals accumulate and interlock, mechanically disrupting and segmenting the gel matrix. Such microstructural damage severely weakens interparticle connectivity, ultimately causing macroscopic strength reduction [89]. This mechanism also explains the strength regression at high DG dosages: as short-prismatic AFt content progressively increases with curing age, its destructive effects on the gel matrix intensify.

### 2.7. Limits and Future Perspectives

This study compared the enhancing effects of three chemical activators and two expansive agents on aeolian sand stabilized with industrial solid wastes based geopolymer (ASIG) through strength tests and a series of characterization techniques examining reaction products and microstructure, while also exploring the underlying mechanisms. Although the findings offer certain insights and guidance for the engineering application of stabilized aeolian sand, several limitations remain, suggesting potential avenues for future research.

Firstly, the maximum curing age considered for the ASIG in this study was only 28 days, and the associated mechanistic analysis was limited to the influence of additives on reaction products and pore structure during short- to medium-term curing. Although the 28-day strength is the most commonly used evaluation metric in foundational, subgrade, and structural engineering, from a scientific perspective, it remains unresolved whether the effects of these additives on ASIG follow the same trends over longer periods. Furthermore, it is unclear whether prolonged curing could lead to adverse effects on the microstructure—such as over-expansion or water absorption—caused by the expansive agents used.

Additionally, from an engineering standpoint, ASIG will inevitably be exposed to destructive desert environmental factors after construction, including aridity, high temperatures, and UV radiation. Under these conditions, whether the additives considered herein would still exert the influences reported in this study, or whether the mechanisms of influence might alter, are critical questions that warrant focused investigation in future studies.

On the other hand, the ultimate value of the investigated ISWs and additives lies in their practical engineering application. Therefore, understanding how chemical activators and expansive agents affect the workability of stabilized aeolian sand is highly important for related projects—an aspect not covered in this study. Future research could focus on whether the addition of alkali activators shortens the setting time of ISWs-stabilized aeolian sand, and how the inclusion of expansive agents alters durability indicators such as shrinkage, swilling rate, and disintegration resistance. Research on the latter could also provide complementary insights into the mechanisms and effective stress contributions of expansive agents, thereby enabling a deeper understanding of stress transfer patterns within stabilized aeolian sand foundations under working loads and facilitating the development of theoretical results such as constitutive models.

## 3. Conclusions

This study comparatively investigated the enhancement effects of three chemical activators (NaOH, Ca(OH)_2_, CaCl_2_) and two expansive agents (DG and BE) on the strength of ASIG using UCS as the evaluation index. Through XRD, TG/DTG, SEM/EDS, MIP, and pH tests, the influences of these additives on phase composition, micro-morphology, and pore structure of ASIG were analyzed. The main conclusions can be summarized as follows:

1. In terms of strength enhancement, expansive agent including BE and DG proved significantly more effective than traditional alkaline activators (NaOH, Ca(OH)_2_, CaCl_2_) in improving the UCS of aeolian sand-based geopolymer. While chemical activators enhanced 28-day strength by approximately 10–15% at optimal concentrations, DG and BE led to increases of 52.6% and 71.3%, respectively, primarily by effectively filling interparticle pores. However, excessive dosage of all additives ultimately leads to a reduction in strength.
2. Regarding the strengthening mechanism, the granular and low-water-content nature of aeolian sand necessitates reliance on physical pore filling rather than interparticle bonding. Chemical activators promoted gel and crystalline phase formation (e.g., AFt, HAP), but excessive use coarsened the pore structure or consumed reactive components. In contrast, DG facilitated dense AFt formation and morphology refinement, while BE compacted the matrix through expansion—both effectively reducing harmful pores and optimizing pore size distribution.
3. The findings of this study highlight that employing expansion-based additives offers a more effective strategy for aeolian sand stabilization than reliance on chemical activation alone. BE compensates for the insufficient pore-filling capability of gels, resulting in a denser microstructure and higher ultimate strength, thereby providing a new material selection and methodological direction for stabilization of aeolian sand.

## 4. Materials and Methods

### 4.1. Materials

The aeolian sand sampling location is situated in the central Taklimakan Desert, Xinjiang, China (77°46′ E, 39°36′ N, elevation 1130 m). Characterized by perennial aridity, the region receives less than 100 mm annual precipitation and experiences persistent wind speeds averaging 5.9 m/s, resulting in over 80% mobile dunes. Samples were obtained from a depth of 1.5–2 m below the ground surface and immediately stored in light-proof sealed bags. Laboratory testing determined the physical properties of the aeolian sand listed in Table 2, with shear strength parameters (cohesion c and internal friction angle φ) measured using a quadruple direct shear apparatus. The sand exhibits negligible silt/clay content, extremely low moisture content, and high porosity, contributing to its loose structure and minimal interparticle bonding.

Among the four main ISWs used to prepare geopolymers, GGBFS with grade S95 was provided by Liyang Mineral Powder Plant in Hebei, China. Coarse SS, PS, and CS were collected from a steel mills, phosphate chemical plants, and acetylene plants in Hebei, China, respectively. Laboratory processing involved ball milling with ball/material ratio as 1:1 and speed as 512 r/min followed by sieving through No. 8 mesh.

Solid activators with analytical grade were supplied by Sinopharm Chemical Reagent Co., Ltd. The expansive agents DG and Ca-based BE were collected from coal-fired power plants and bentonite mines in Xinjiang, China, respectively.

### 4.2. Specimens Design

This study primarily investigates the effects of three activators, NaOH, Ca(OH)_2_, and CaCl_2_, and two expansive agents, DG and BE, on strength of ASIG. The ISWs proportion was previously optimized through the three chemical moduli of cement clinker and mixture design experiments, determined as SS:GGBFS:PS:CS = 5:35:20:40, corresponding to the highest UCS of stabilized aeolian sand specimens. The total ISWs dosage was fixed at 11%, a typical level for stabilizing non-cohesive soils and sandy materials and a standard dosage stipulated in Chinese subgrade specifications [91,92].

In the previous study [14], an orthogonal experimental design was employed to investigate the effect of moisture content—ranging from 3% to 15%—on the strength of cement-stabilized aeolian sand. The results revealed that 9% moisture content represented a critical threshold for strength development; beyond this value, a decrease in specimen strength was observed. Similar conclusions have been drawn in the literature [92]. This phenomenon is likely attributable to the fact that this specific moisture content simultaneously satisfies the required water-to-cement ratio for the added cement and approximates the optimum moisture content for the aeolian sand itself [4,8]. Consequently, to ensure a fair and equivalent comparison between the effectiveness of the geopolymer investigated in this study and that of cement in stabilizing aeolian sand, the specimen moisture content was likewise determined to be 9%.

The masses of dry aeolian sand, ISWs, and water added to each specimen were kept equal to control variables, ensuring that only the dosage of the external additives varied. The mass of dry aeolian sand was first determined to be 306.30 g by multiplying the measured dry density of the aeolian sand by the specimen volume. Subsequently, the total mass of ISWs added was calculated as 33.7 g. According to proportions established in previous studies, the respective masses of the four ISWs were calculated as follows: SS: 1.68 g, GGBFS: 11.79 g, PS: 6.74 g, and CS: 13.48 g. The mass of distilled water added was 27.56 g. Five dosage levels for each additive were considered. Given the high CaO content in the ISWs used, the dosage levels of alkali activators were selected based on previous studies concerning the incorporation of such activators in cement or high-calcium geopolymer systems [93,94,95,96]. The design experiment series are detailed in Table 3, where the dosages of the chemical activators are expressed as molar concentration (M, calculated based on the total water content) and those of the expansive agents are quantified as a mass fraction of dry aeolian sand (%). 27 specimens were designed for each curing age. With three ages under consideration (3, 14, and 28 days), this resulted in a total of 81 specimens. Furthermore, to minimize experimental error, 3 parallel specimens were prepared for each proportion and curing condition. The final reported strength for each specimen type represents the average value obtained from these three identical parallel specimens.

### 4.3. Specimens Preparation

The preparation of the specimens complies with the American standard ASTM 5102-22 [97]. All ISWs were placed in an oven and dried at 105 °C for 6 h, and then transferred to the bowl of a planetary mixer and mixed at low speed until uniform color was achieved, followed by the addition of aeolian sand dried and passed through No. 20 sieve with continued mixing until homogeneity was reestablished. For the control groups, distilled water was added to the mixing bowl in a fine stream. Additives with high solubility (NaOH and CaCl_2_) were first dissolved in distilled water, and corresponding solution were prepared using a 100 mL standard volumetric flask for calibration before being introduced into the mixing bowl via a fine stream. Poorly soluble or insoluble additives were directly added to the mixing bowl and blended, after which distilled water was incorporated. Following additives addition, high-speed mixing was conducted for an additional 5 min. The mixture was then loaded into stainless steel molds (50 mm diameter × 100 mm height) in three sequential layers, each compacted with 15 strikes using a small rammer and scarified with a knife to roughen the surface. The filled molds were sealed with plastic film and cured at room temperature for three days. After demolding, the specimens were transferred to a standard ambient temperature and humidity curing chamber for further curing under controlled conditions: a temperature of 23 ± 2 °C and a relative humidity exceeding 90%. And the specimens were wrapped in plastic film to prevent excessive carbonation by isolating them from atmospheric CO_2_.

### 4.4. Test Method

UCS was measured using an electro-hydraulic universal testing machine equipped with a 10 kN load sensor (accuracy: 0.001 kN) and a displacement sensor with 0.01 mm resolution. Upon reaching the curing age, specimens were centrally positioned on the machine bottom plate. A preload of 0.1 mm/min was applied until the load sensor registered 0.05 kN to ensure proper contact, after which the loading rate was adjusted to 0.08 mm/min until specimen failure.

A part of fragmented specimen was ground into powder and freeze-dried to terminate hydration reactions, followed by phase characterization. Mineralogical composition was analyzed using a D8 ADVANCE X-ray diffractometer (Bruker, Hong Kong) with Cu Kα radiation generated at 60 kV and 80 mA. Scanning range was set to 5°~80°, with step size of 0.02°, and scan rate of 0.5 s/step. Thermogravimetric analysis (TGA) was performed using an SDT Q600 synchronous thermal analyzer (TA Instruments Inc., New Castle, DE, USA) to evaluate chemical composition and thermal stability of stabilized aeolian sand. Samples were equilibrated at ambient temperature for 30 min prior to heating from 25 °C to 800 °C at 10 °C/min under nitrogen atmosphere, with continuous recording of mass loss and derivative thermogravimetric (DTG) curves.

Microstructural morphology of stabilized aeolian sand was examined using an SU8220 field emission scanning electron microscope (FE-SEM, HITACHI High-tech Co., Tokyo, Japan). To mitigate charging artifacts caused by low conductivity of materials, specimens were coated with a 10 nm Au-Pd layer using a Desk II sputter coater (Denton, UK). Imaging was conducted under high vacuum at an accelerating voltage of 5 kV, and the secondary electron detector was employed. Meanwhile, energy dispersive X-ray spectroscopy (EDS) was performed using back-scattered electron detectors at an accelerating voltage of 15 kV for elemental composition determination.

Mercury intrusion porosimetry (MIP) was performed using an AutoPore V 9620 porosimeter (Micromeritics, Norcross, GA, USA) to quantitatively characterize the microporous structure of ASIG. Fragments (10 mm diameter) obtained from the central region of specimens were analyzed. The measurements covered detectable pore diameters between 0.001 µm and 300 µm.

Considering that MIP measurements of pores in the gel matrix on particle surfaces may be influenced by interparticle capillary pores, a SEM image-based identification methodology was employed to determine the matrix porosity. The procedure involves: (1) identifying relatively intact hydration products encapsulating sand particles under SEM, (2) capturing high-magnification images of these regions, (3) using ImageJ 1.54 software to identify pores and calculate porosity.

The pH of ASIG was determined in accordance with ASTM D4972 [98]. The ground and freeze-dried specimen was mixed with distilled water at a mass ratio of 1:1, homogenized using a magnetic stirrer for 5 min under plastic film-sealed conditions, and subsequently equilibrated at 23 °C (±2 °C) for 1 h. The supernatant pH was then measured using a PHS-3C pH meter (INESA, China), which was calibrated with buffer solutions (pH = 6.86 and 9.18 at 23 °C).

**Figure 17:**
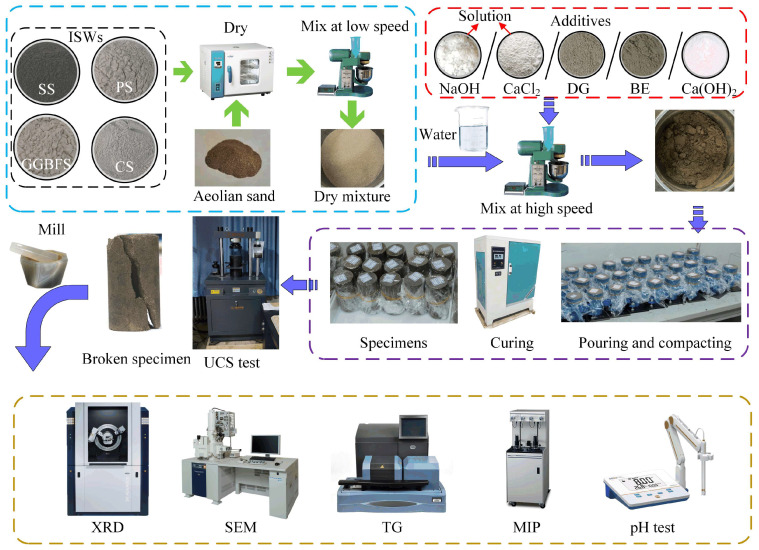
Specimen preparation process and the testing procedures.

The specimen preparation process and the testing procedures used are shown in Figure 17.

## Figures and Tables

**Table 1:** Chemical compositions of ISWs and expansive agents.

Materials	SiO_2_	CaO	Fe_2_O_3_	Al_2_O_3_	MgO	SO_3_	Na_2_O	K_2_O	P_2_O_5_
Steel slag (SS)	17.18	30.01	29.1	8.43	4.41	0.62	0	0.01	0.87
Ground granulated blast furnace slag (GGBFS)	30.07	38.22	0.88	16.50	8.33	2.08	0.51	0.86	0.02
Phosphorus slag (PS)	44.89	43.97	0.01	3.88	3.72	0	0.05	0.84	1.28
Carbide slag (CS)	2.72	70.35	1.03	0.89	0.62	0	0	0	0.02
Desulfurized gypsum (DG)	0.60	48.25	0.57	0.60	1.17	38.51	0.52	1.72	0.02
Bentonite (BE)	66.58	3.35	2.42	14.57	2.56	0	2.37	1.39	0.11

**Table 2:** Basic properties of aeolian sand [90].

Properties	Characteristics	Method
Specific gravity, *G*_s_	2.64	Pycnometer
Natural water content, *w*_0_ (%)	2.50	Oven drying
Natural density, *ρ* (g/cm^3^)	1.56	Cutting ring
Initial void ratio, *e*_0_	0.81	-
Saturation, *S*_r_ (%)	8.14	-
Cohesion, *c* (kPa)	0.26	Direct shear
Internal friction angle *φ* (°)	33.70	Direct shear
Sand fraction (>0.075 mm) (%)	98.01	laser size analysis
Silt fraction (0.002–0.075 mm) (%)	1.99	laser size analysis
Clay fraction (<0.002 mm) (%)	-	laser size analysis

**Table 3:** Experiments design.

Series	Specimen Name	Additives	Dosage	Mass of Additives (g)	Curing Ages (Days)
Activators	SH0.15C*i*	NaOH	0.15 M	0.165	3, 14, 28
	SH0.30C*i*		0.30 M	0.331	
	SH0.45C*i*		0.45 M	0.496	
	SH0.60C*i*		0.60 M	0.661	
	SH0.75C*i*		0.75 M	0.827	
	CH0.10C*i*	Ca(OH)_2_	0.10 M	0.204	
	CH0.20C*i*		0.20 M	0.408	
	CH0.30C*i*		0.30 M	0.613	
	CH0.40C*i*		0.40 M	0.817	
	CH0.50C*i*		0.50 M	1.021	
	CL0.05C*i*	CaCl_2_	0.05 M	0.153	
	CL0.10C*i*		0.10 M	0.306	
	CL0.15C*i*		0.15 M	0.459	
	CL0.20C*i*		0.20 M	0.612	
	CL0.25C*i*		0.25 M	0.765	
Expansive agents	DG0.4C*i*	Desulfurized gypsum	0.40%	1.225	
	DG1.0C*i*		1.00%	3.063	
	DG1.6C*i*		1.60%	4.901	
	DG2.2C*i*		2.20%	6.739	
	DG2.8C*i*		2.80%	8.576	
	BE0.4C*i*	Bentonite	0.40%	1.225	
	BE1.0C*i*		1.00%	3.063	
	BE1.6C*i*		1.60%	4.901	
	BE2.2C*i*		2.20%	6.739	
	BE2.8C*i*		2.80%	8.576	
Control	BLC*i*	-	-	-	
	CEC*i*	-	-	-	

Footnote: The first two letters in each specimen name represent the abbreviation of the additive: SH denotes NaOH, CH represents Ca(OH)_2_, CL indicates CaCl_2_, DG stands for desulfurized gypsum, BE refers to bentonite, BL represents the control group without any additives, and CE indicates the control group using an equivalent dosage of cement to stabilize aeolian sand. The suffix C*i* indicates a curing period of *i* days for the specimen. For example, SH0.15C28 designates a specimen with 0.15 M NaOH addition cured for 28 days.

**Figure A1:**
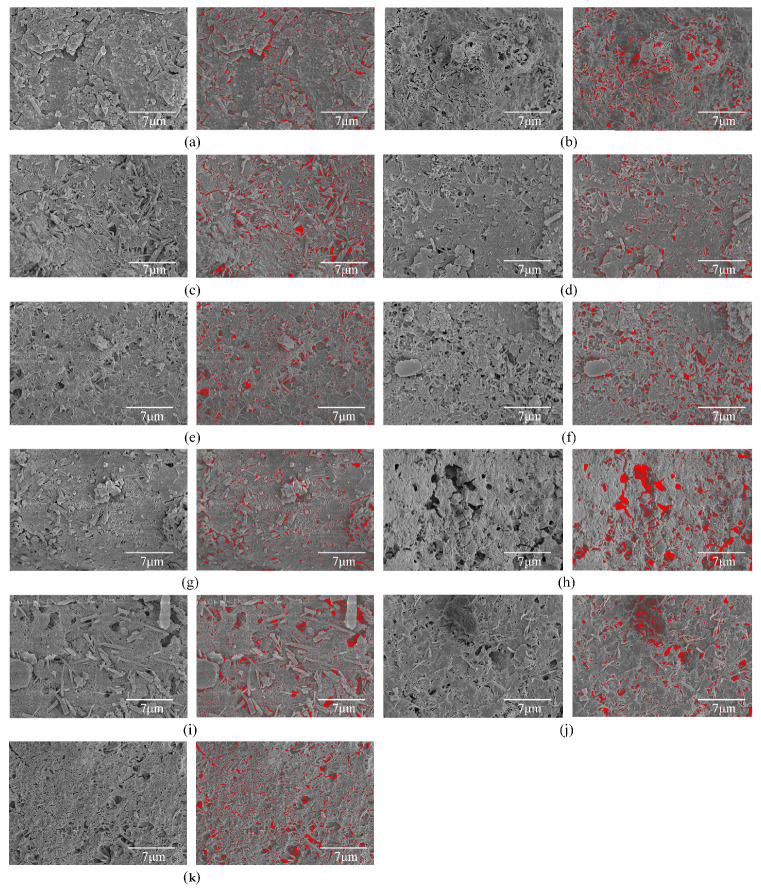
SEM images utilized for pore identification and corresponding results: (**a**) SH0.45C28; (**b**) SH0.75C28; (**c**) CH0.1C28; (**d**) CH0.4C28; (**e**) CL0.15C28; (**f**) CL0.3C28; (**g**) DG2.2C28; (**h**) DG2.8C28; (**i**) BE1.6C28; (**j**) BE2.8C28; (**k**) BLC28.

## Data Availability

The raw data supporting the conclusions of this article will be made available by the authors upon request.

## Appendix A

### SEM Images Utilized for Pore Identification and Corresponding Results

The regions marked in red represent the identified pores. Since pore identification by the software is dependent on the detection threshold as well as the color of the image itself, the brightness, contrast, detection threshold, and magnification were kept consistent for all SEM images.
